# Supplementary material for: HIVprotI: an integrated web based platform for prediction and design of HIV proteins inhibitors
Source: J Cheminform. 2018 Mar 9;10:12. doi: 10.1186/s13321-018-0266-y (PMC5845081; doi:10.1186/s13321-018-0266-y)
Supplement: Supplementary file 2 — Additional file 2. Source code of HIVProtI web server. [file 13321_2018_266_MOESM2_ESM.zip › HIVprotI_Source-code/hivprotI/weka-3-6-6/doc/weka/filters/unsupervised/attribute/Add.html]

Add


---


|  |  |  |
| --- | --- | --- |
| **Overview** \| **Package** \| **Class** \| **Tree** \| **Deprecated** \| **Index** \| **Help** \| **Weka's home** | |  |
| **PREV CLASS**   **NEXT CLASS** | **FRAMES**    **NO FRAMES**     **All Classes** |  |
| SUMMARY: NESTED \| FIELD \| CONSTR \| METHOD | DETAIL: FIELD \| CONSTR \| METHOD |  |


---


## weka.filters.unsupervised.attribute Class Add

```
java.lang.Object
  weka.filters.Filter
      weka.filters.unsupervised.attribute.Add
```

**All Implemented Interfaces:**: java.io.Serializable, CapabilitiesHandler, OptionHandler, RevisionHandler, StreamableFilter, UnsupervisedFilter

---

``` public class Add extends Filter implements UnsupervisedFilter, StreamableFilter, OptionHandler ```

An instance filter that adds a new attribute to the dataset. The new attribute will contain all missing values.


Valid options are:

```
 -T <NUM|NOM|STR|DAT>
  The type of attribute to create:
  NUM = Numeric attribute
  NOM = Nominal attribute
  STR = String attribute
  DAT = Date attribute
  (default: NUM)
```

```
 -C <index>
  Specify where to insert the column. First and last
  are valid indexes.(default: last)
```

```
 -N <name>
  Name of the new attribute.
  (default: 'Unnamed')
```

```
 -L <label1,label2,...>
  Create nominal attribute with given labels
  (default: numeric attribute)
```

```
 -F <format>
  The format of the date values (see ISO-8601)
  (default: yyyy-MM-dd'T'HH:mm:ss)
```

**Version:**
:   $Revision: 5543 $

**Author:**
:   Len Trigg (trigg@cs.waikato.ac.nz)

**See Also:**: Serialized Form

---

| **Field Summary** | |
| --- | --- |
| `static Tag[]` | `TAGS_TYPE`             the attribute type. |


**Constructor Summary**

`Add()`


| **Method Summary** | |
| --- | --- |
| `java.lang.String` | `attributeIndexTipText()`             Returns the tip text for this property. |
| `java.lang.String` | `attributeNameTipText()`             Returns the tip text for this property. |
| `java.lang.String` | `attributeTypeTipText()`             Returns the tip text for this property |
| `java.lang.String` | `dateFormatTipText()`             Returns the tip text for this property. |
| `java.lang.String` | `getAttributeIndex()`             Get the index of the attribute used. |
| `java.lang.String` | `getAttributeName()`             Get the name of the attribute to be created. |
| `SelectedTag` | `getAttributeType()`             Gets the type of attribute to generate. |
| `Capabilities` | `getCapabilities()`             Returns the Capabilities of this filter. |
| `java.lang.String` | `getDateFormat()`             Get the date format, complying to ISO-8601. |
| `java.lang.String` | `getNominalLabels()`             Get the list of labels for nominal attribute creation. |
| `java.lang.String[]` | `getOptions()`             Gets the current settings of the filter. |
| `java.lang.String` | `getRevision()`             Returns the revision string. |
| `java.lang.String` | `globalInfo()`             Returns a string describing this filter. |
| `boolean` | `input(Instance instance)`             Input an instance for filtering. |
| `java.util.Enumeration` | `listOptions()`             Returns an enumeration describing the available options. |
| `static void` | `main(java.lang.String[] argv)`             Main method for testing this class. |
| `java.lang.String` | `nominalLabelsTipText()`             Returns the tip text for this property. |
| `void` | `setAttributeIndex(java.lang.String attIndex)`             Sets index of the attribute used. |
| `void` | `setAttributeName(java.lang.String name)`             Set the new attribute's name. |
| `void` | `setAttributeType(SelectedTag value)`             Sets the type of attribute to generate. |
| `void` | `setDateFormat(java.lang.String value)`             Set the date format, complying to ISO-8601. |
| `boolean` | `setInputFormat(Instances instanceInfo)`             Sets the format of the input instances. |
| `void` | `setNominalLabels(java.lang.String labelList)`             Set the labels for nominal attribute creation. |
| `void` | `setOptions(java.lang.String[] options)`             Parses a given list of options. |

**Methods inherited from class weka.filters.Filter**

`batchFilterFile, batchFinished, filterFile, getCapabilities, getOutputFormat, isFirstBatchDone, isNewBatch, isOutputFormatDefined, makeCopies, makeCopy, numPendingOutput, output, outputPeek, toString, useFilter, wekaStaticWrapper`

**Methods inherited from class java.lang.Object**

`equals, getClass, hashCode, notify, notifyAll, wait, wait, wait`

**Field Detail**

### TAGS\_TYPE

```
public static final Tag[] TAGS_TYPE
```

:   the attribute type.


**Constructor Detail**

### Add

```
public Add()
```


**Method Detail**

### globalInfo

```
public java.lang.String globalInfo()
```

:   Returns a string describing this filter.

    :   **Returns:**: a description of the filter suitable for displaying in the explorer/experimenter gui

---


### listOptions

```
public java.util.Enumeration listOptions()
```

:   Returns an enumeration describing the available options.

    :   **Specified by:**: `listOptions` in interface `OptionHandler`
    :   **Returns:**: an enumeration of all the available options.

---


### setOptions

```
public void setOptions(java.lang.String[] options)
                throws java.lang.Exception
```

:   Parses a given list of options.


    Valid options are:

    ```
     -T <NUM|NOM|STR|DAT>
      The type of attribute to create:
      NUM = Numeric attribute
      NOM = Nominal attribute
      STR = String attribute
      DAT = Date attribute
      (default: NUM)
    ```

    ```
     -C <index>
      Specify where to insert the column. First and last
      are valid indexes.(default: last)
    ```

    ```
     -N <name>
      Name of the new attribute.
      (default: 'Unnamed')
    ```

    ```
     -L <label1,label2,...>
      Create nominal attribute with given labels
      (default: numeric attribute)
    ```

    ```
     -F <format>
      The format of the date values (see ISO-8601)
      (default: yyyy-MM-dd'T'HH:mm:ss)
    ```

    :   **Specified by:**: `setOptions` in interface `OptionHandler`
    :   **Parameters:**: `options` - the list of options as an array of strings **Throws:**: `java.lang.Exception` - if an option is not supported

---


### getOptions

```
public java.lang.String[] getOptions()
```

:   Gets the current settings of the filter.

    :   **Specified by:**: `getOptions` in interface `OptionHandler`
    :   **Returns:**: an array of strings suitable for passing to setOptions

---


### getCapabilities

```
public Capabilities getCapabilities()
```

:   Returns the Capabilities of this filter.

    :   **Specified by:**: `getCapabilities` in interface `CapabilitiesHandler` **Overrides:**: `getCapabilities` in class `Filter`
    :   **Returns:**: the capabilities of this object **See Also:**: `Capabilities`

---


### setInputFormat

```
public boolean setInputFormat(Instances instanceInfo)
                       throws java.lang.Exception
```

:   Sets the format of the input instances.

    :   **Overrides:**: `setInputFormat` in class `Filter`
    :   **Parameters:**: `instanceInfo` - an Instances object containing the input instance structure (any instances contained in the object are ignored - only the structure is required). **Returns:**: true if the outputFormat may be collected immediately **Throws:**: `java.lang.Exception` - if the format couldn't be set successfully

---


### input

```
public boolean input(Instance instance)
```

:   Input an instance for filtering. Ordinarily the instance is processed
    and made available for output immediately. Some filters require all
    instances be read before producing output.

    :   **Overrides:**: `input` in class `Filter`
    :   **Parameters:**: `instance` - the input instance **Returns:**: true if the filtered instance may now be collected with output(). **Throws:**: `java.lang.IllegalStateException` - if no input format has been defined.

---


### attributeNameTipText

```
public java.lang.String attributeNameTipText()
```

:   Returns the tip text for this property.

    :   **Returns:**: tip text for this property suitable for displaying in the explorer/experimenter gui

---


### getAttributeName

```
public java.lang.String getAttributeName()
```

:   Get the name of the attribute to be created.

    :   **Returns:**: the new attribute name

---


### setAttributeName

```
public void setAttributeName(java.lang.String name)
```

:   Set the new attribute's name.

    :   **Parameters:**: `name` - the new name

---


### attributeIndexTipText

```
public java.lang.String attributeIndexTipText()
```

:   Returns the tip text for this property.

    :   **Returns:**: tip text for this property suitable for displaying in the explorer/experimenter gui

---


### getAttributeIndex

```
public java.lang.String getAttributeIndex()
```

:   Get the index of the attribute used.

    :   **Returns:**: the index of the attribute

---


### setAttributeIndex

```
public void setAttributeIndex(java.lang.String attIndex)
```

:   Sets index of the attribute used.

    :   **Parameters:**: `attIndex` - the index of the attribute

---


### nominalLabelsTipText

```
public java.lang.String nominalLabelsTipText()
```

:   Returns the tip text for this property.

    :   **Returns:**: tip text for this property suitable for displaying in the explorer/experimenter gui

---


### getNominalLabels

```
public java.lang.String getNominalLabels()
```

:   Get the list of labels for nominal attribute creation.

    :   **Returns:**: the list of labels for nominal attribute creation

---


### setNominalLabels

```
public void setNominalLabels(java.lang.String labelList)
```

:   Set the labels for nominal attribute creation.

    :   **Parameters:**: `labelList` - a comma separated list of labels **Throws:**: `java.lang.IllegalArgumentException` - if the labelList was invalid

---


### attributeTypeTipText

```
public java.lang.String attributeTypeTipText()
```

:   Returns the tip text for this property

    :   **Returns:**: tip text for this property suitable for displaying in the explorer/experimenter gui

---


### setAttributeType

```
public void setAttributeType(SelectedTag value)
```

:   Sets the type of attribute to generate.

    :   **Parameters:**: `value` - the attribute type

---


### getAttributeType

```
public SelectedTag getAttributeType()
```

:   Gets the type of attribute to generate.

    :   **Returns:**: the current attribute type.

---


### dateFormatTipText

```
public java.lang.String dateFormatTipText()
```

:   Returns the tip text for this property.

    :   **Returns:**: tip text for this property suitable for displaying in the explorer/experimenter gui

---


### getDateFormat

```
public java.lang.String getDateFormat()
```

:   Get the date format, complying to ISO-8601.

    :   **Returns:**: the date format

---


### setDateFormat

```
public void setDateFormat(java.lang.String value)
```

:   Set the date format, complying to ISO-8601.

    :   **Parameters:**: `value` - a comma separated list of labels

---


### getRevision

```
public java.lang.String getRevision()
```

:   Returns the revision string.

    :   **Specified by:**: `getRevision` in interface `RevisionHandler` **Overrides:**: `getRevision` in class `Filter`
    :   **Returns:**: the revision

---


### main

```
public static void main(java.lang.String[] argv)
```

:   Main method for testing this class.

    :   **Parameters:**: `argv` - should contain arguments to the filter: use -h for help


---


|  |  |  |
| --- | --- | --- |
| **Overview** \| **Package** \| **Class** \| **Tree** \| **Deprecated** \| **Index** \| **Help** \| **Weka's home** | |  |
| **PREV CLASS**   **NEXT CLASS** | **FRAMES**    **NO FRAMES**     **All Classes** |  |
| SUMMARY: NESTED \| FIELD \| CONSTR \| METHOD | DETAIL: FIELD \| CONSTR \| METHOD |  |


---
